# Discrepancies in Self-reporting of Bride Kidnapping in Kyrgyzstan: Concealment or misperception?

**DOI:** 10.1007/s12110-025-09500-1

**Published:** 2025-09-13

**Authors:** Narhulan Halimbekh, Olympia L. K. Campbell, Yishan Xie, Anar Erjan, Anna Dmitrieva, Almagul Aisarieva, Zhamila Zhalieva, Damira Toktorova, Cholpon Kabylovna Sooronbaeva, Ruth Mace

**Affiliations:** 1 Department of Anthropology, University College London, London, UK; 2 Institute for Advanced Study in Toulouse, University of Toulouse Capitole, Toulouse, France; 3 Faculty of Asian and Middle Eastern Studies, University of Oxford, Oxford, UK; 4 School of English, Communication and Philosophy, Cardiff University, Cardiff, UK; 5 Department of Linguistics, Kyrgyz National University, Bishkek, Kyrgyzstan; 6 Technical School of Innovation, the American University of Central Asia, Bishkek, Kyrgyzstan; 7 Faculty of World Languages and Cultures, Osh State University, Osh, Kyrgyzstan; 8 Department of Psychology, Pedagogy and Humanitarian Science, Yssyk-Kul State University after name of K.Tynystanov, Karakol, Kyrgyzstan

**Keywords:** Forced marriage, Bride kidnapping, Kyrgyzstan, Misreport, Misperception

## Abstract

Bride kidnapping, where Women are abducted for marriage, persists in Kyrgyzstan despite being illegal. Although it is estimated that up to one-third of marriages in Kyrgyzstan result from abduction, the true prevalence of this practice is unknown. Estimates are based on self-reporting of a practice that has become illegal. Here we examine whether there are sex and intergenerational differences in this reporting, that reflect a changing legal and social environment that might influence the self-reporting of bride kidnapping marriage. Using data from 468 participants in two Kyrgyz villages collected through 2023, this study examines self-reporting discrepancies in kidnap marriages among married couples. Significant differences were found in how husbands and wives report their marriages: husbands often describe the marriages as consensual, while wives see them as non-consensual. These discrepancies show a convergence over time, with couples married more recently agreeing on the marriage type. Furthermore, fathers often reported their son’s marriages as consensual, while the sons themselves reported them as non-consensual, highlighting a generational divide. Our findings suggest a normative transformation driven by cohort replacement, where evolving attitudes toward consent erode the cultural mechanisms sustaining bride kidnapping. This offers insight into the evolutionary dynamics of such gender-biased harmful practices, highlighting how legal reforms and societal pressures reshape perceptions over time.

**Supplementary Information:**

The online version contains supplementary material available at 10.1007/s12110-025-09500-1.

## Introduction

### Bride Kidnapping: Context and Challenges

Bride kidnapping, also known as bride capture or marriage by abduction, is a type of forced marriage where a man forcibly takes a woman for marriage (Barnes, 1999). Bride kidnapping has been documented in many societies across the world, notably in regions of Africa (e.g., South Africa), the Caucasus (e.g., Georgia), Central Asia (e.g., Kyrgyzstan, Kazakhstan), and parts of Southeast Asia (e.g., Indonesia) (Buckner, 2018; Kleinbach & Salimjanova, 2007; Rice, 2018; United Nations Population Fund & United Nations Children’s Fund, 2020; Voell, 2012).

Bride kidnapping is deeply embedded in complex socio-cultural traditions, gender roles, and hierarchical social structures (Hofmann & Chi, 2022; Muldoon & Casabonne, 2017; Sataeva, 2017). In many patriarchal societies, bride kidnapping functions as a mechanism for reinforcing male dominance, facilitating strategic familial alliances, or circumventing economically burdensome marriage practices, such as bride prices or dowry payments (Werner, 2009; Werner et al., 2018). From an evolutionary perspective, forced marriage, including bride kidnapping, has been explored through the lens of reproductive strategies and sexual conflict: in environments characterised by limited resources, intense male-male competition, or constrained female choice, bride kidnapping may emerge as a coercive mating strategy whereby men seek to increase reproductive success by restricting women’s autonomy in partner selection (Buss, 2023; Buss & Duntley, 2011; Holub & Vance, 2022). Understanding bride kidnapping’s cultural roots and underlying evolutionary mechanisms is essential, not only to grasp its historical persistence but also to address its modern challenges, particularly concerning human rights and gender equality.

The practice of bride kidnapping varies widely, but generally takes both consensual and non-consensual forms (Ayres, 1974; Barnes, 1999). Consensual kidnapping can include both “mock bride kidnapping” and “ceremonial bride kidnapping” (Amsler & Kleinbach, 1999; Ayres, 1974; Kleinbach et al., 2005). In the former, although appearing to resist her captors, the woman has secretly agreed to or even planned the proceeding. In the latter, a ritual performance takes place with the full consent and knowledge of members of both parties. In non-consensual bride kidnapping, however, only men are exerting choice. Non-consensual bride kidnapping violates women’s rights, potentially resulting in emotional trauma, physical harm, forced physical relations, social isolation, restricted opportunities in education, employment, and personal autonomy, adverse health outcomes, cultural tensions such as conflicts between traditional practices and modern values, and legal challenges (Ali et al., 2009; Borbieva, 2012; Human Rights Watch, 2019; Kim & Karioris, 2021; Samad, 2010; Werner, 2009). While its prevalence has declined in many areas due to legal reforms, education, advocacy, and shifting attitudes toward gender equality, non-consensual bride kidnapping persists in some regions despite being illegal (Ahearn, 2001; Borbieva, 2012; Lee, 2013; McLaren, 2001; Morewitz, 2019).

However, accurately estimating the prevalence and nature of bride kidnapping poses significant methodological challenges, especially given its sensitive nature. Researchers studying such topics often rely on self-report measures, which are vulnerable to various biases. According to social norm theory, individuals’ behaviours and beliefs are shaped by what they perceive to be common (descriptive norms) and socially approved (injunctive norms) within their communities (Bicchieri, 2005). In this context, biased reporting may reflect attempts to conform to perceived injunctive norms (what people think is acceptable), even if those perceptions are inaccurate or shifting. Social desirability bias and normalisation bias are particularly relevant in this regard, which not only affects individual responses but also contribute to the misperception of social norms, making harmful practices appear more or less prevalent than they actually are (Krumpal, 2013; Lawson et al., 2024).

Understanding the extent and nature of reporting biases in cultural practices like bride kidnapping is crucial for accurately assessing their prevalence and designing effective interventions. This study aims to investigate whether social desirability bias or normalisation bias affects how individuals report their marriage experiences. Our methodology compares direct self-reports from married couples with indirect reports from their parents and siblings, analysing discrepancies within and across generations. Specifically, we aim to address three research questions: (1) What is the overall prevalence and nature of bride kidnapping across the study period? (2) To what extent do social desirability bias or normalisation bias contribute to differences between husbands’ and wives’ reports of marriage consensuality, and how do these compare with discrepancies between participants and their family members (parents and siblings)? (3) How have husband-wife reporting differences evolved over time, and what do these trends suggest about normative change?

We examine this phenomenon in Kyrgyzstan, where bride kidnapping remains prevalent despite legal prohibitions. Drawing on social norm theory and cultural evolution theory, we hypothesise that reporting patterns will reflect both gendered differences in perception and evolving cultural norms across generations. Specifically, we hypothesise that: (1) Husbands will report their marriages and their children’s bride kidnappings as more consensual than wives, seeking to align with legal expectations and maintain social approval. (2) Reporting discrepancies will decrease among younger cohorts and across generations, reflecting a normative shift toward recognising non-consensual practices, driven by cohort replacement and evolving cultural values. This study connects individual perceptions to cultural change, laying the groundwork for future ecological analyses of bride kidnapping’s persistence.

### Reporting Biases: Social Desirability Bias and Normalisation Bias

Social desirability bias reflects individuals altering their responses, either consciously through impression management or unconsciously via self-deception, to conform to socially acceptable standards, leading to misreporting (Dutton & Hemphill, 1992; Krumpal, 2013; Zerbe & Paulhus, 1987). Impression management involves deliberately adjusting answers to align with external expectations (Kempf Leonard, 2005; Zerbe & Paulhus, 1987). For example, a study on woman empowerment shows that respondents may intentionally exaggerate their approval of women’s empowerment to appear socially progressive, even when privately holding different views (Lawson et al., 2021). Self-deception, by contrast, occurs when individuals unconsciously rationalise their actions to maintain a positive self-view or self-esteem (Bicchieri, 2005; Dutton & Hemphill, 1992; Kempf Leonard, 2005). For instance, in communities practicing female genital mutilation (FGM), some practitioners believe that FGM protects girls’ moral integrity and enhances marriageability, despite the clear medical and psychological harm it causes (Gupta, 2013; van Veen et al., 2018).

In contrast, normalisation bias occurs when individuals genuinely perceive socially problematic or harmful behaviours as normal or harmless due to cultural or societal conditioning (Lawson et al., 2024; Tankard & Paluck, 2016). Unlike social desirability bias, which involves distortion of truth, normalisation bias results in truthful but culturally conditioned responses. For instance, in communities where intimate partner violence (IPV) is culturally accepted, individuals genuinely believe these behaviours are normal or non-harmful (Abdelshahid & Campbell, 2015; Gibson et al., 2022).

Both biases often reflect gender-specific patterns tied to social roles, resulting in gender-specific norm perception, thus misreport intentionally or unintentionally (Krumpal, 2013; Lawson et al., 2024; Tourangeau & Yan, 2007). For example, men often exaggerated their number of sexual partners to align with masculine ideals, whereas women underreported to conform with social expectations of modesty (Alexander & Fisher, 2003). In the case of emotional support, men unconsciously underreport their struggles as they believe such behaviour inflicts masculinity, whereas women sought help more openly (Addis & Mahalik, 2003; Iwamoto et al., 2012). In the context of harmful practices, these biases could potentially distort prevalence estimates, hinder accurate research, and perpetuate harmful norms by masking their full scope and impact.

Moreover, these biases are not fixed; they can shift over time through cohort replacement, which refers to the gradual transformation of cultural norms, values, and practices as one generation succeeds another (Kiley & Vaisey, 2020; Ochoa & Vaisey, 2024; Vaisey & Lizardo, 2016). This generational shift may reflect cultural evolutionary processes, as younger cohorts, characterised by greater acceptance and support, gradually replace older ones, societal norms evolve (Ekstam, 2023). Considering the substantial changes in social norms concerning gender roles and traditional values across many countries in recent decades (Beisheeva et al., 2022; UN Women, 2024), it is possible that similar dynamics could be occurring with bride kidnapping. It is unknown whether the prevalence of bride kidnapping is affected by social desirability bias, misperception, cohort replacement, or a combination of them all.

### Bride Kidnapping in Kyrgyzstan

We focus on bride kidnapping in Kyrgyzstan, a post-Soviet country located in Central Asia. Bride kidnapping (known as “*ala kachuu*”) has been prevalent in Kyrgyzstan for decades, perhaps centuries. The exact origins of this practice have been a subject of debate. Some scholars suggest that bride kidnapping is a traditional Kyrgyz practice and was originally a form of consensual elopement, by which the bride and the groom could avoid having to seek their parents’ approval. Others argue that prior to the Soviet Union, bride kidnapping was rare (Bagirova, 2021; Kleinbach & Salimjanova, 2007). Instead, they propose that the poverty and unemployment that resulted from compulsory collectivisation made it financially difficult to marry, leading men to secure marriage via forceful kidnapping. A third group emphasise that bride kidnapping is a longstanding historical tradition among the Kyrgyz people, which is believed to have re-emerged with the process of de-Sovietisation and the construction of an ethno-national identity by the independent Kyrgyz state after the collapse of Soviet Union (Anagnost, 1997; Kandiyoti, 2009; Kim & Karioris, 2021; Werner, 2009). This narrative also gained traction among nationalists and traditionalists, who sought to endorse such customs as revitalised traditions (Gal & Kligman, 2000; Werner, 2009). Whatever the history, bride kidnapping is deeply rooted in traditional norms and social expectations in Kyrgyzstan, and was, until recently, widely accepted.

The practice of bride kidnapping in Kyrgyzstan typically involves a young woman being taken by force or deception by a group of men, including the intended groom. These men could be people she knows, or they can be strangers. Sometimes female friends of the groom or bride may also assist in deceiving the woman to facilitate her kidnapping. The kidnap could happen in various locations such as the woman’s home, school, workplace, or from the street. Upon arrival at the man’s home, the woman is put in a room and surrounded by the groom’s female relatives, who will welcome the kidnapped woman with warmth and hospitality, while at the same time exerting pressure on her to “agree” to the marriage proposal. A head scarf placed over her head signals acceptance of the marriage (Kim & Karioris, 2021; Kleinbach & Salimjanova, 2007; Werner, 2009). Since it is an honour-based society, a woman who resists (“girl who returned home”) often encounters social stigma from the community, as her actions are perceived as bringing disgrace and dishonour upon herself, her family, and the groom’s family (Borbieva, 2012; Kim & Karioris, 2021; Werner, 2009). Given the possibility of rape, people often assume that kidnapped women may no longer be virgins, leading to them being viewed as unsuitable for marriage in the future (Human Rights Watch, 2006; Kim & Ukueva, 2020). Often parents advise kidnapped daughters to stay to maintain the family honour (Human Rights Watch, 2006; Kim & Ukueva, 2020). It is estimated that only 8 to 17% of bride kidnappings do not result in marriage (Amsler & Kleinbach, 1999; Kleinbach et al., 2005).

Since the 2000 s, there has been increased attention on the issue of bride kidnapping (UNDP Kyrgyzstan, 2022; United Nations, 2022). The criminal code of Kyrgyzstan has undergone multiple amendments since 1997 (see more details in Table 1), with the most recent amendment, which entered into force on December 1, 2021, stating that the abduction of a person for marriage is punishable by imprisonment for 5–7 years (The Ministry of Justice of Kyrgyz Republic, 2024). However, prosecutions are rare and many villages in Kyrgyzstan are *de facto* ruled by the councils of elders (“*aqsaqal*”) following customary law, which may override state legal system (Beyer, 2006; Handrahan, 2004; Muldoon & Casabonne, 2017). Councils of elders often do not take bride kidnapping seriously, and in many cases, members of the council are invited to the wedding and encourage the bride and her family to accept the marriage (Human Rights Watch, 2006). In 2021, of 560 cases of registered abduction and forced marriage of Women, 82 cases were taken to court while 460 cases were dismissed (UNDP Kyrgyzstan, 2022).

**Table 1:** Key historical and legal developments in Kyrgyzstan

Year	Event	Description
1926	Kyrgyz Autonomous Soviet Socialist Republic	Formation of the Kyrgyz Autonomous Soviet Socialist Republic within the Russian Soviet Federative Socialist Republic.
1936	Kyrgyz Soviet Socialist Republic	Elevation to the status of a full Soviet Socialist Republic.
1991	Independence from the Soviet Union	Declaration of independence from the Soviet Union.
1992	Joined United Nations	Upon joining the United Nations in 1992, Kyrgyzstan accepted the obligations of the UN Charter, which includes adherence to the principles outlined in the Universal Declaration of Human Rights.
1997	Introduction of the first criminal code specifically addressing bride kidnapping, making it a punishable offense under Kyrgyz law.	Article 154 (3) Kidnapping of a person under the age of sixteen for the purpose of de facto marriage, shall be punished by deprivation of liberty for a period of three to seven years. Article 155 When forcing a woman to marry or to continue living together after marriage, or abducting her against her will for marriage, as well as preventing a woman from marrying, shall be fined in the amount of one hundred to two hundred of the minimum wage or shall be sentenced to imprisonment for a term of up to five years.
2007	Amendment to the Criminal Code against bride kidnapping: the imprisonment or restriction of liberty punishment for kidnapping Women under 16 remained unchanged, whereas, for kidnapping adults is reduced to three years.	Article 154 (2) Kidnapping of a person under sixteen years of age for entering into actual marital relations is punishable by imprisonment for a term of three to seven years. (As amended by the Law of the Kyrgyz Republic dated June 25, 2007 No. 91) Article 155 Forcing a woman into marriage or continuation of marriage cohabitation or abduction for marriage against her will, and equally preventing a woman from entering into marriage shall be punished by a fine in the amount of one hundred to two hundred monthly calculation indexes or restriction of freedom for a term of up to three years. (As amended by the Law of the Kyrgyz Republic dated June 25, 2007 No. 91)
2013	Amendment to the Criminal Code against bride kidnapping: Change in age from 16 to 17. Increase in imprisonment or restriction of liberty punishments for kidnapping girls under 17 to 5 to 10 years, and for kidnapping adult Women from 3 years to 5 to 7 years. It is also important to notice here that, the 2013 amendment had deleted the monetary option as punishment. As you can see in the law clauses before, kidnapping adult Women is punished by either an extensive fine or imprisonment, but this is no longer the case after the 2013 amendment.	Article 154 (2) Abduction of a person under seventeen years of age for the purpose of entering into de facto marital relations shall be punishable by imprisonment for a term of five to ten years. (As amended by the Law of the Kyrgyz Republic dated January 25, 2013 No. 9) Article 155 (2) Abduction of a woman for marriage against her will shall be punishable by imprisonment for a term of five to seven years. (As amended by the Law of the Kyrgyz Republic dated January 25, 2013 No. 9)
2024	The Latest version	Article 172 1. Kidnapping of a person for marriage shall be punishable by imprisonment for a term of five to seven years. 2. Abduction of a child for entering into actual marital relations or for marriage shall be punishable by imprisonment for a term of seven to ten years.

## Materials and Methods

### Study Site

Fieldwork was conducted in two distinct villages situated in Tong District, Kyrgyz Republic. One is located beside a lake, while the other one is located approximately 25 km inland, nestled in a mountainous region. These villages were selected for the study due to their strong connections with our local gatekeepers, the residents’ general openness to social surveys, and the continued practice of traditional Kyrgyz cultural customs, including bride kidnapping.

### Data Collection

Data collection spanned 2023. Seven female research assistants who were proficient in Kyrgyz and knowledgeable about regional social norms, conducted face-to-face interviews with adult men and women in the villages. Five were of Kyrgyz ethnicity, while the remaining two, though not ethnically Kyrgyz, were fluent in Kyrgyz and had a strong understanding of local culture. Local village assistants accompanied them during interviews to help build rapport and streamline the process.

The survey consisted of two main components: a household-level demographic survey and an individual-level marital history questionnaire. For the demographic survey, one adult per household (typically the head or another available adult) provided, with written informed consent, information about all long-term resident household members, including name, sex, age, residency status, relationship to the head, education, employment, and the household’s primary income source.

The marital history questionnaire was administered individually to consenting adults who were long-term village residents. Participants provided details about their marriage, including marriage year and marriage type (specifically whether kidnapping was involved in their marriage, and if yes, whether it was a consensual kidnapping, where both bride and groom agreed to the arrangement in advance, or a non-consensual kidnapping). Where possible, interviews were conducted in private, comfortable settings, such as participants’ homes or workplaces. To minimise sampling bias from interviewing only those available during initial visits, research assistants revisited households when residents were unavailable, ensuring broader coverage of eligible participants.

In addition to self-reporting their marriage history, each participant also provided information on their parents’ and siblings’ marriage types. Because some participants’ family members (parents, siblings, or children) also resided in the same villages and participated in our survey, we were able to cross-validate these self-reports with data independently reported by their kin. This allowed us to construct both individual self-reports and external validations provided by parents or siblings.

Across the villages, 509 consenting adults out of 800 were interviewed for the marital history questionnaire. Reasons for non-participation included time constraints due to work or family obligations, reluctance to discuss sensitive marital histories, or absence during the fieldwork. Of those interviewed, some responses were excluded due to reliability concerns: nine due to potential response bias from interviews conducted in the presence of family members and 32 for incomplete marital history data. The final dataset included 468 participants (255 Women, 213 men), representing 171 married couples. Among these, 51 individuals had corresponding marriage reports from their mothers, 38 from their fathers, 38 from their sisters, and 64 from their brothers. When multiple siblings provided reports for a participant, all were included in the analysis to preserve data richness.

### Ethics Statement

This research received approval from the UCL (University College London) Research Ethics Committee (reference no: 0499/004). Permission to research in Kyrgyzstan was granted as the study was conducted in collaboration with the Aigine Cultural Research Centre (ACRC, https://aigine.kg/).

Prior to participation, each individual received a detailed information sheet explaining the project’s aims, methods, and the types of data to be collected. This sheet explicitly highlighted the voluntary nature of participation, confidentiality protocols, and participants’ rights, including the freedom to skip any uncomfortable questions or withdraw from the study at any time without any repercussions. Interviews proceeded only after obtaining signed consent from each participant.

Given the sensitive nature of the research, particularly concerning forced or non-consensual marriage experiences, interviews were conducted privately. All participants were provided with contact information for the Association of Crisis Centres of Kyrgyzstan, should they need psychological, medical, or legal assistance.

### Statistical Analysis

To examine discrepancies between married couples’ self-reported marriage information, we constructed a contingency table to display the frequencies of consistent and discrepant reports between husbands and wives. A Chi-squared test was then performed to assess whether the observed differences in reporting were statistically significant. The same approach was applied to compare participants’ self-reported marriage information with the information provided by their parents and siblings.

To analyse temporal changes in reported marriage types among married couples, we developed a multinomial logistic regression model with the husband’s and wife’s reported marriage types (non-consensual kidnap marriage is coded as 0, consensual kidnap marriage is coded as 1, no-kidnap marriage is coded as 2) as the response variable. The explanatory variable was the marriage year cohort, grouped into ten-year intervals. Due to small sample sizes, cohorts before 1980 and after 2010 were merged, resulting in five cohorts in total. The model also includes an interaction term with respondent gender to capture potential sex differences in reporting patterns across cohorts. Based on this model, we used estimated marginal means (EMMs) to calculate the predicted probability of each marriage type by gender within each cohort.

A second multinomial model was constructed to assess spousal reporting discrepancies, the response variable indicated whether husband’s and wife’s reported marriage types were consistent or not (Consistent: both spouses agree and provide the same reports, coded as 0; HRMC: husband reports more consensual marriage types, coded as 1; or WRMC: wife reports more consensual marriage types, coded as 2). For the explanatory variable, the same cohort variable was used, except that cohorts before 1990 and after 2000 were merged due to limited sample size, resulting in three cohorts in total. Predicted probabilities for each discrepancy category by cohort were also computed using estimated marginal means. All figures were generated using the ggplot2 package, and statistical analyses were conducted in R version 3.5.1 (R Core Team, 2018).

## Results

### Demographic and Socioeconomic Information of Studied Regions

According to our demographic survey, both villages are medium-sized, each comprising fewer than 200 long-term resident households. The predominant residents in both villages are Kyrgyz, with high levels of educational attainment. The primary sources of income are farming and livestock. Both villages follow a patrilocal system, where wives relocate to their husbands’ villages after marriage. As a result, nearly all wives in these villages originate from other communities. Divorce is extremely rare in these villages. Islam is the main religion, though the degree of religious practice varies widely among villagers, ranging from those who never practice to those who engage in religious activities more than once a week (more details can be found in Table 2).

**Table 2:** Demographic and socioeconomic characteristics of the study villages in Kyrgyzstan

Variables	Village 1	Village 2
Number of households	179	91
Number of adults registered	544	256
Adult sex ratio (male%)	0.50	0.47
Average age	47.5	44.9
Main ethnicity	Kyrgyz (98%)	Kyrgyz (100%)
Education (%)		
No education	2.00	0.82
Primary	1.77	1.65
Secondary	49.67	65.02
Technical school or college	24.39	13.17
Higher education	22.17	19.34
Main income source (%)		
No income	0	1.23
Farming	75.06	70.08
Business employment	9.56	2.05
Government job	6.29	11.89
Pension	8.62	12.7
Other	0.47	3.28
Number of divorces	9	7
Average marital dispersal distance of females (km)	133.05	197

### Descriptive Information of Bride Kidnapping Marriage in Studied Regions

**Fig. 1:**
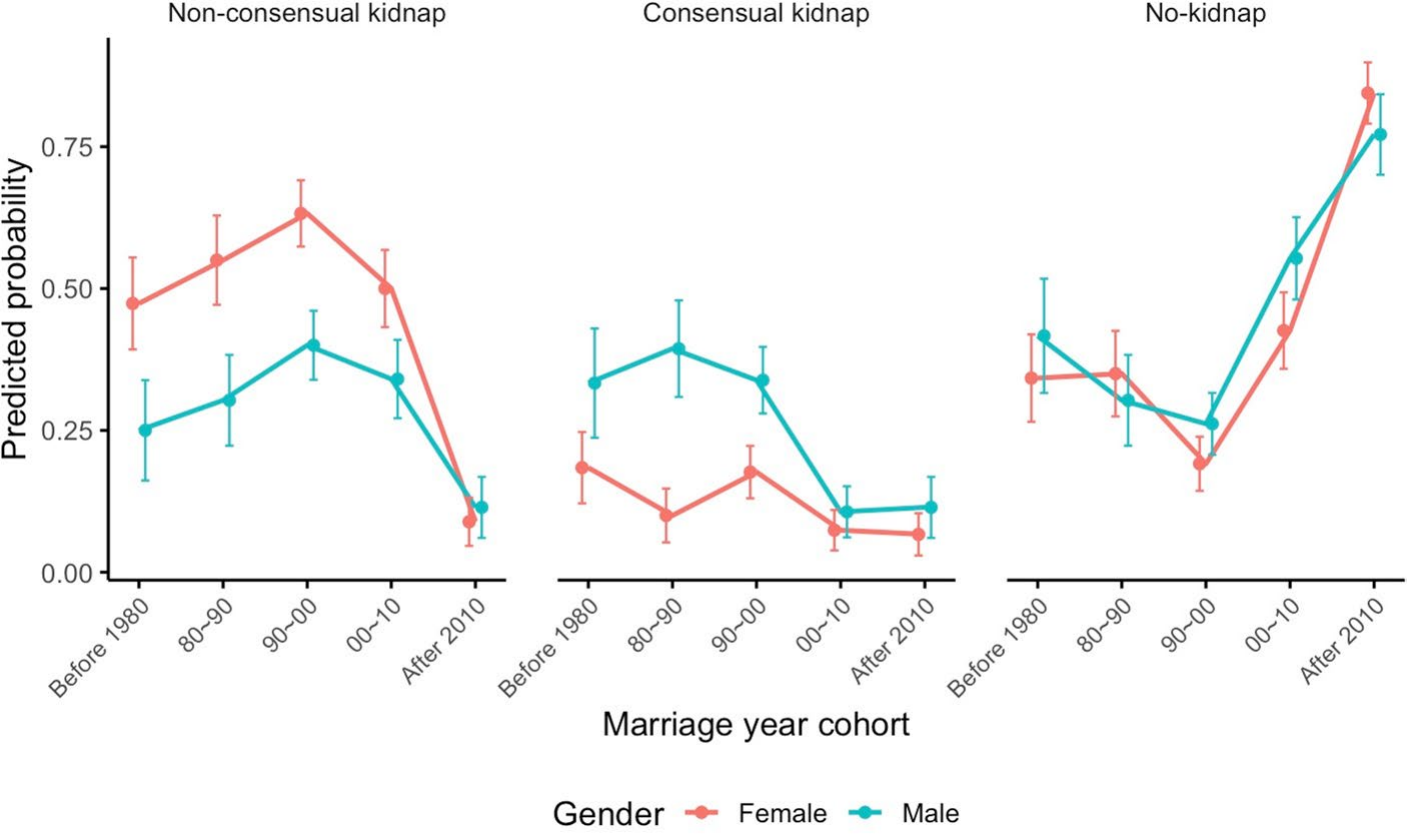
The temporal change of predicted probability of reporting different marriage types (non-consensual kidnap, consensual kidnap and no-kidnap) in male and female participants. Points depict the mean predicted probability of each marriage type, and error bars indicating standard errors

Across all participants in the two villages, marriage types show consistent proportions: 57% of Women and 56% of men reported being married through either consensual or non-consensual kidnapping, with a notable gender difference in non-consensual cases (45.8% of Women vs. 30.5% of men; Table 3). Temporal trends reveal broadly similar patterns between men’s and women’s reports: both indicate a peak in non-consensual kidnap marriages in the 1990 s (probabilities of 0.63 for Women and 0.40 for men), followed by a substantial decline after 2010 (0.09 for Women, 0.11 for men). Correspondingly, reports from both genders show a significant rise in no-kidnap marriages since 2000, reaching probabilities of 0.84 for Women and 0.77 for men after 2010 (Fig. 1; full details provided in Table S1).

**Table 3:** Summary of marriage types reported by males and females across different marriage year cohorts in two villages

	Village 1		Village 2		Overall	
	Females	Males	Females	Males	Females	Males
Number of participants	164	129	91	84	255	213
Non-consensual kidnap	71 (43.3%)	32 (24.8%)	46 (50.5%)	33 (39.3%)	117 (45.8%)	65 (30.5%)
Before 1980	9	3	9	3	18	6
1980–1990	14	2	8	8	22	10
1990–2000	28	18	15	8	43	36
2000–2010	15	4	12	12	27	16
After 2010	3	2	1	2	4	4
Unreported	2	3	1	0	3	3
Consensual kidnap	17 (10.4%)	34 (26.4%)	14 (15.4%)	20 (23.8%)	31 (12.2%)	54 (25.4%)
Before 1980	4	3	3	5	7	8
1980–1990	2	11	2	2	4	13
1990–2000	8	15	4	7	12	22
2000–2010	1	3	3	2	4	5
After 2010	1	0	2	4	3	4
Unreported	1	2	0	0	1	2
No-kidnap	76 (46.3%)	63 (48.8%)	31 (34.1%)	31 (36.9%)	107 (42.0%)	94 (44.1%)
Before 1980	11	7	2	3	13	10
1980–1990	7	7	7	3	14	10
1990–2000	10	11	3	6	13	17
2000–2010	17	19	6	7	23	26
After 2010	25	15	13	12	38	27
Unreported	6	4	0	0	6	4

### Disparities and Temporal Change in Reported Bride Kidnapping Information in Married Couples

Within husband-wife dyads, temporal trends in marriage types align broadly with overall population patterns described in Sect. 3.2, yet distinct disparities emerge clearly between husbands and wives. Across all cohorts, wives consistently reported higher probabilities of non-consensual kidnap marriages compared to their husbands. This disparity was most pronounced during the 1990 s period, though it decreased after 2000 as both spouses converged toward reporting low levels of non-consensual kidnappings. Conversely, husbands generally reported higher rates of consensual kidnapping, particularly in earlier cohorts. Nevertheless, by recent cohorts, both genders reported similarly low probabilities for consensual kidnapping. Reports of no-kidnap marriages exhibited a clear upward trajectory from the 1990 s onward in both husbands and wives (Figure S1, Table S2-a&b).

**Fig. 2:**
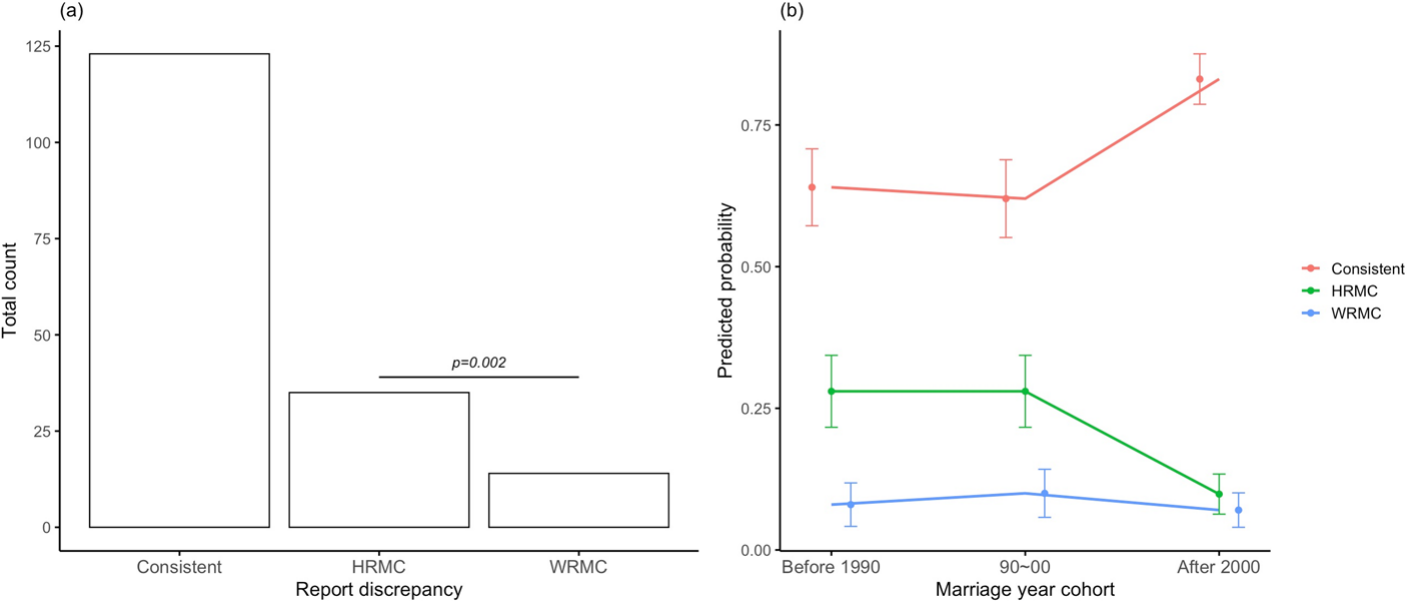
(**a**) Frequency of consistent and discrepant reporting of marriage types between husbands and wives, categorised as Consistent: both spouses agree and provide the same reports, HRMC: husband reports more consensual marriage types, or WRMC: wife reports more consensual marriage types. Chi-squared tests were used to examine differences between discrepant groups, with results displayed in the panel. (**b**) Temporal trends in the predicted probability of each reporting category across marriage year cohorts, with points representing mean probabilities and error bars indicating standard errors

Direct comparisons within individual husband-wife pairs further underscore these gender differences. Inconsistencies in marriage-type reporting were common, with husbands frequently characterising their marriages as more consensual (either as consensual kidnappings or no-kidnap marriages) than their wives did (Fig. 2a; see contingency table in Table S3-a). These reporting discrepancies declined over time, particularly in husbands, reflecting a growing alignment between spouses in recent marriage cohorts (Fig. 2b; Table S2-c&d).

### Disparities in Reported Bride Kidnapping Information Across Self-reports, Parental Reports, and Sibling Reports

**Fig. 3:**
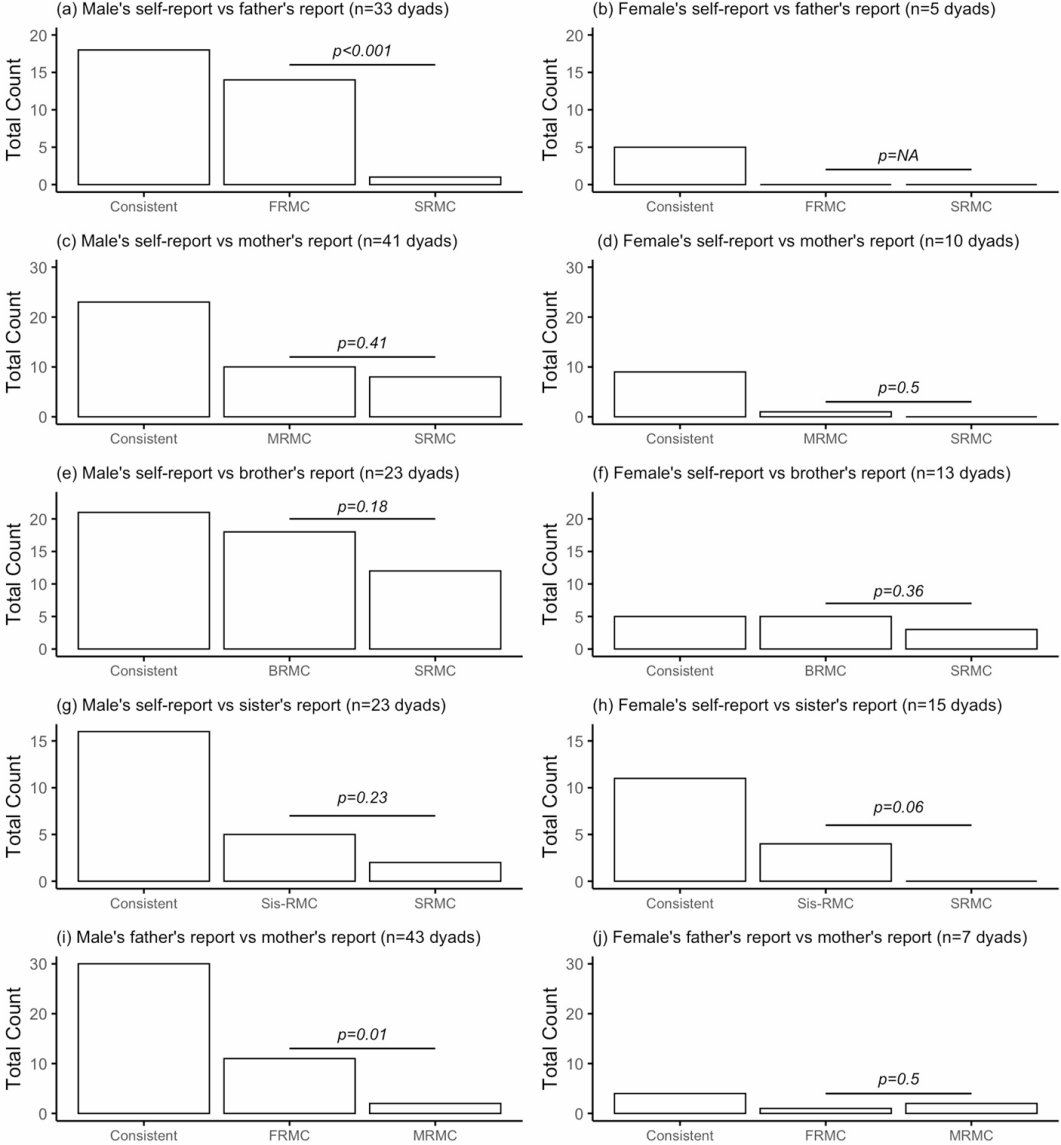
(**a-h**) show the frequency of consistent and discrepant reporting of marriage types between male and female participants and their family members (fathers, mothers, brothers, and sisters). Panels (**a-d**) display comparisons with parents, while panels (**e-h**) show comparisons with siblings. Each panel presents the frequency of “Consistent” reports (where both parties agree on the marriage type) and discrepant reports, categorised as follows: FRMC (Father Reports More Consensual Marriage), MRMC (Mother Reports More Consensual Marriage), BRMC (Brother Reports More Consensual Marriage), Sis-RMC (Sister Reports More Consensual Marriage), and SRMC (Self Reports More Consensual Marriage). (**i-j**) show the frequency of consistent and discrepant reporting of marriage types between participants’ fathers and mothers, separated by participant’s gender. Chi-squared tests were used to examine differences between discrepant groups, with results displayed on each panel

Inconsistent reports on participant’s marriage types were observed between participants’ self-reports and those provided by family members (see contingency table in Table S3-b). While all family members tended to report participants’ marriages as more consensual than participants themselves, no significant patterns were found in the discrepancies with mothers (Fig. 3c-d), brothers (Fig. 3e-f), or sisters (Fig. 3g-h), regardless of the participants’ gender. However, a significant difference was observed in discrepancies between participants’ self-reports and their fathers’ reports, which occurred exclusively between fathers and sons: fathers were significantly more likely to report their sons’ marriages as more consensual marriage types (Fig. 3a).

We also identified discrepancies between fathers’ and mothers’ reports of their children’s marriage types. Fathers are more likely than mothers to report their sons’ marriage as more consensual marriage types (Fig. 3i), although this difference was not observed in daughters (Fig. 3j).

## Discussion

Our study reveals significant patterns in how bride kidnapping is reported among rural Kyrgyz communities, with important implications for understanding both the prevalence of this practice and the sociocultural mechanisms that sustain it.

### Prevalence and Temporal Trends of Bride Kidnapping

Our study shows that the kidnap marriage is still common in rural Kyrgyzstan, however, has been in major deline. Among the marriages reported by male participants and those reported by female participants, each considered independently, over 50% involved bride kidnapping, whether consensual or non-consensual (Table 3). The likelihood of self-reported kidnappings among current residents peaked in the late 1990 s, followed by a marked decline, whereas the likelihood of self-reported no-kidnap marriage has been on the increase, and the patterns are similar in both males and females (including the married couples). The overall temporal trends observed in our study regions align with patterns reported in previous studies (Nedoluzhko & Agadjanian, 2015; Werner, 2009; Werner et al., 2018), which suggest that bride kidnapping began increasing during the Soviet era, intensified following Kyrgyzstan’s independence in 1991, and has shown a gradual decline in recent years. The significant decrease in kidnapping prevalence after 2010 corresponds with strengthened legal sanctions against the practice and their enforcement.

### Gender Disparities in Reporting Marriage Experiences

A central finding of our study is the systematic gender difference in how marriages are described. Husbands consistently report lower rates or frequency of non-consensual kidnapping compared to their wives. One possible explanation for husbands’ underreporting of non-consensual kidnapping is social desirability bias. As bride kidnapping has become increasingly criminalised in Kyrgyzstan, men may feel compelled to characterise their marriages as consensual to align themselves with current social and legal norms. The progressive strengthening of legal sanctions against bride kidnapping has likely heightened the stigma associated with admitting involvement in forced marriages.

Normalisation bias may also provide a more nuanced explanation for the observed gender differences. Rather than intentionally misrepresenting their experiences, men may genuinely perceive kidnapping differently from women because of cultural conditioning. In Kyrgyzstan’s patriarchal context, men typically hold positions of decision-making power, which may lead men to interpret coercive elements of bride kidnapping within culturally accepted frameworks of courtship or tradition (Ismailbekova, 2016; Porreca, 2025). Furthermore, the post-Soviet revival of traditional Kyrgyz identity has repositioned bride kidnapping as a marker of cultural authenticity (Kleinbach & Salimjanova, 2007; Werner, 2009), potentially normalising such practices in men’s perceptions, while women’s experiences remain defined by their lack of consent. The consistent gender differences observed across multiple family relationships further reinforce the normalisation bias explanation. Husbands and wives differ not only in reporting their own marriage experiences, but fathers and mothers also exhibit similar discrepancies when describing their children’s marriages. This recurring pattern across family roles suggests that the difference likely stems from deeply embedded cultural frameworks rather than intentional misreporting.

### Evidence of Normative Change

We also show a notable convergence in reporting discrepancies among more recent marriages, with couples from later cohorts more likely to agree on marriage types. Despite the dramatic decline in kidnapping prevalence in recent cohorts may naturally lead to fewer opportunities for disagreement about their consensual nature (see Table 3), several aspects of our data suggest that normative change is occurring. First, the timing of changes in both reporting patterns and kidnapping prevalence corresponds with legal reforms, suggesting that formal sanctions may be influencing both behaviour and perceptions (Lundberg, 2021). Second, the probability of reporting discrepancies has decreased over time.

, we would expect reporting discrepancies to widen in recent cohorts rather than narrow. Third, all data were collected contemporaneously, all participants, regardless of marriage timing, responded to the same interviewers under the same normative conditions. This methodological consistency means that observed differences across marriage cohorts cannot be attributed to variations in interview conditions or changing social pressures during data collection. Rather, they likely reflect genuine historical changes in how marriages were formed and perceived. The convergence in reporting indicates a shifting normative landscape in which younger generations are developing shared understandings of consent and coercion in marriage formation. This shift appears to be primarily driven by changes in men’s perceptions, as younger men demonstrate greater alignment with women’s characterisations of marriage consent than their predecessors.

### Generational Differences and Cohort Replacement

Beyond differences between husbands and wives, our findings reveal significant generational patterns in reporting marriage information. The most striking example is that fathers report their sons’ marriages as more consensual than the sons themselves report, while no such pattern exists between mothers and their children or between siblings. This intergenerational pattern defies expectations based on simple social desirability explanations. If contemporary social pressures were driving reporting tendencies, we would expect younger men to be more susceptible to such pressures than their fathers, who were married when bride kidnapping carried less stigma. Instead, we find sons more willing than their fathers to acknowledge non-consensual aspects of marriage formation, suggesting authentic perceptual differences rather than differential impression management.

These findings align with research on cohort replacement as a mechanism of normative change (Kiley & Vaisey, 2020; Ochoa & Vaisey, 2024), suggesting that generational turnover rather than individual attitudinal shifts drives the evolution of cultural practices. The generational divide among men likely reflects competing frameworks for understanding marital consent in post-Soviet Kyrgyzstan. Older men may view kidnapping marriages as fundamentally traditional and implicitly consensual, whereas younger men are more inclined to emphasise individual choice and the importance of explicit consent (Handrahan, 2004; Human Rights Watch, 2006; Porreca, 2025).

The absence of similar generational patterns among women suggests that women’s perceptions of consent in marriage have remained more consistent across generations, potentially because women’s experiences of non-consensuality are more directly tied to their bodily autonomy and personal agency, which may be less susceptible to reinterpretation through changing cultural frameworks.

### Conclusion, Implications and Limitations

Our study reveals that discrepancies in how bride kidnapping is reported are not merely due to misreporting, but reflect deeper gendered differences in perception shaped by cultural norms. Men, especially older generations, tend to describe marriages as more consensual than women do, likely due to normalisation bias rooted in patriarchal social structures. We also find that these discrepancies diminish among younger cohorts. Recent generations, particularly younger men, show greater alignment with women in reporting non-consensual elements of marriage, indicating a shift in normative frameworks regarding consent. This convergence supports the idea that cultural transformation is occurring through cohort replacement rather than individual attitude change.

Our findings offer important insights into the cultural evolution of gender-biased practices like bride kidnapping. By tracing how reporting discrepancies shift across generations, our study reveals how norms surrounding consent and coercion are changing over time. The weakening of normalisation bias signals an evolutionary shift in gendered perception, possibly driven by legal reforms and changing social environments. Understanding these perceptual changes helps explain how harmful practices decline: not solely through external sanctions, but through internalised shifts in cultural meaning. This perspective is essential for behavioural and evolutionary approaches to studying how traditions persist, adapt, or erode under shifting selective pressures.

Our study has limitations. Due to patrilocal residence patterns, we were very limited in our ability to collect perspectives from the wives’ natal families, which may have offered additional insight into marriage experiences. The retrospective nature of our data also restricts our ability to capture evolving perceptions as they occur. Additionally, the data were collected from a limited geographic area, constraining the generalisability of our findings and potentially not reflecting the full complexity of bride kidnapping trends across Kyrgyzstan. Future research could build on this work by incorporating broader kinship perspectives, using longitudinal designs, and expanding data collection to more regions to better examine the evolutionary dynamics that sustain or diminish bride kidnapping practices over time.

## Supplementary Information

Below is the link to the electronic supplementary material.

Supplementary Material 1

## Declarations

### Data Accessibility

Data are available in the following Github repository: https://github.com/narhulan29/Misreporting.git.
