# Supplementary material for: Discrepancies in Self-reporting of Bride Kidnapping in Kyrgyzstan: Concealment or misperception?
Source: Hum Nat. 2025 Sep 13;36(3):382–402. doi: 10.1007/s12110-025-09500-1 (PMC12644216; doi:10.1007/s12110-025-09500-1)
Supplement: Supplementary file 1 — Supplementary Material 1 [file 12110_2025_9500_MOESM1_ESM.docx]

**SUPPLEMENTARY MATERIAL**

**
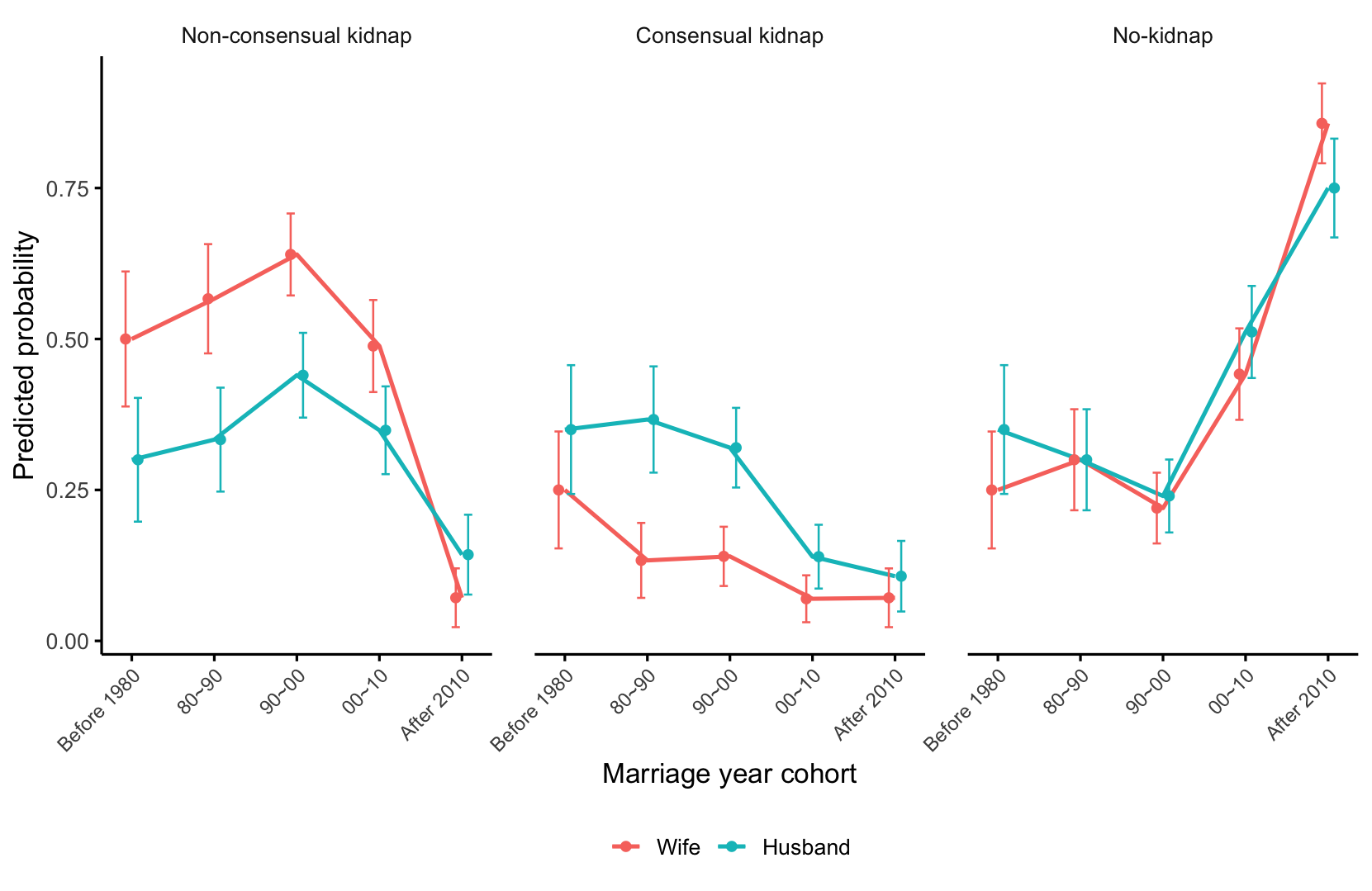
**

**Figure S1**. Temporal trends in the predicted probability of marriage types (non-consensual kidnap, consensual kidnap, or no-kidnap) reported by husbands and wives across marriage year cohorts, with points representing mean probabilities and error bars indicating standard errors.

**Table S1. (a)** Results of a multinomial logistic regression model examining the interaction between marriage year cohort and participant’s gender on the likelihood of reporting different marriage types (reference category: non-consensual kidnap, woman and pre-1980).

| *Response* | *Predictors* | *Odds Ratios* | *Standard error* | *p* |
| --- | --- | --- | --- | --- |
| Consensual kidnap | Intercept | 0.39 | 0.17 | **0.04** |
|  | 1980s | 0.47 | 0.33 | 0.28 |
|  | 1990s | 0.72 | 0.4 | 0.55 |
|  | 2000s | 0.38 | 0.27 | 0.17 |
|  | After 2010 | 1.93 | 1.71 | 0.46 |
|  | Male | 3.43 | 2.4 | 0.08 |
|  | 1980s ×male | 2.09 | 2.05 | 0.45 |
|  | 1990s×male | 0.88 | 0.73 | 0.88 |
|  | 2000s×male | 0.62 | 0.63 | 0.63 |
|  | After 2010×male | 0.39 | 0.49 | 0.45 |
| No-kidnap | Intercept | 0.72 | 0.26 | 0.37 |
|  | 1980s | 0.88 | 0.44 | 0.80 |
|  | 1990s | 0.42 | 0.2 | 0.07 |
|  | 2000s | 1.18 | 0.54 | 0.72 |
|  | After 2010 | 13.15 | 8.41 | **<0.001** |
|  | Male | 2.31 | 1.46 | 0.19 |
|  | 1980s ×male | 0.68 | 0.58 | 0.65 |
|  | 1990s×male | 0.94 | 0.72 | 0.93 |
|  | 2000s×male | 0.83 | 0.63 | 0.80 |
|  | After 2010×male | 0.31 | 0.3 | 0.23 |
| Number of participants | 449 | | | |
| R^2^ / R^2^ adjusted | 0.12 / 0.12 | | | |

**Table S1. (b)** Predicted probabilities of marriage types (non-consensual kidnap, consensual kidnap, no-kidnap) by gender and marriage year cohort. Predicted probabilities were estimated based on estimated marginal means, with standard errors shown.

| *Gender* | *Marriage type* | *Marriage year cohort* | *Predicted probability* | *Standard error* |
| --- | --- | --- | --- | --- |
| Females | Non-consensual kidnap | Before 1980 | 0.47 | 0.08 |
|  |  | 1980s | 0.55 | 0.08 |
|  |  | 1990s | 0.63 | 0.06 |
|  |  | 2000s | 0.50 | 0.07 |
|  |  | After 2010 | 0.09 | 0.04 |
|  | Consensual kidnap | Before 1980 | 0.18 | 0.06 |
|  |  | 1980s | 0.10 | 0.05 |
|  |  | 1990s | 0.18 | 0.05 |
|  |  | 2000s | 0.07 | 0.04 |
|  |  | After 2010 | 0.07 | 0.04 |
|  | No-kidnap | Before 1980 | 0.34 | 0.08 |
|  |  | 1980s | 0.35 | 0.08 |
|  |  | 1990s | 0.19 | 0.05 |
|  |  | 2000s | 0.43 | 0.07 |
|  |  | After 2010 | 0.84 | 0.05 |
| Males | Non-consensual kidnap | Before 1980 | 0.25 | 0.09 |
|  |  | 1980s | 0.30 | 0.08 |
|  |  | 1990s | 0.40 | 0.06 |
|  |  | 2000s | 0.34 | 0.07 |
|  |  | After 2010 | 0.11 | 0.05 |
|  | Consensual kidnap | Before 1980 | 0.33 | 0.10 |
|  |  | 1980s | 0.39 | 0.09 |
|  |  | 1990s | 0.34 | 0.06 |
|  |  | 2000s | 0.11 | 0.05 |
|  |  | After 2010 | 0.11 | 0.05 |
|  | No-kidnap | Before 1980 | 0.42 | 0.10 |
|  |  | 1980s | 0.30 | 0.08 |
|  |  | 1990s | 0.26 | 0.05 |
|  |  | 2000s | 0.55 | 0.07 |
|  |  | After 2010 | 0.77 | 0.07 |

**Table S2. (a)** Results of a multinomial logistic regression model examining the interaction between marriage year cohort and spouse gender on the likelihood of reporting different marriage types (reference category: non-consensual kidnap, wife and pre-1980).

| *Response* | *Predictors* | *Odds Ratios* | *Standard error* | *p* |
| --- | --- | --- | --- | --- |
| Consensual kidnap | Intercept | 0.50 | 0.27 | 0.21 |
|  | 1980s | 0.47 | 0.37 | 0.34 |
|  | 1990s | 0.44 | 0.3 | 0.23 |
|  | 2000s | 0.29 | 0.24 | 0.13 |
|  | After 2010 | 2.00 | 2.28 | 0.54 |
|  | Husband | 2.33 | 1.82 | 0.28 |
|  | 1980s × Husband | 2.00 | 2.11 | 0.51 |
|  | 1990s× Husband | 1.42 | 1.35 | 0.71 |
|  | 2000s× Husband | 1.20 | 1.33 | 0.87 |
|  | After 2010× Husband | 0.32 | 0.48 | 0.44 |
| No-kidnap | Intercept | 0.50 | 0.27 | 0.21 |
|  | 1980s | 1.06 | 0.73 | 0.93 |
|  | 1990s | 0.69 | 0.45 | 0.57 |
|  | 2000s | 1.81 | 1.15 | 0.35 |
|  | After 2010 | 23.99 | 22.01 | **0.001** |
|  | Husband | 2.33 | 1.82 | 0.28 |
|  | 1980s × Husband | 0.73 | 0.72 | 0.75 |
|  | 1990s× Husband | 0.68 | 0.63 | 0.68 |
|  | 2000s× Husband | 0.69 | 0.63 | 0.69 |
|  | After 2010× Husband | 0.19 | 0.23 | 0.17 |
| Number of participants | 342 | | | |
| R^2^ / R^2^ adjusted | 0.11 / 0.11 | | | |

**Table S2. (b)** Predicted probabilities of marriage types (non-consensual kidnap, consensual kidnap, no-kidnap) by spouse gender and marriage year cohort. Predicted probabilities were estimated based on estimated marginal means, with standard errors shown.

| *Gender* | *Marriage type* | *Marriage year cohort* | *Predicted probability* | *Standard error* |
| --- | --- | --- | --- | --- |
| Wives | Non-consensual kidnap | Before 1980 | 0.50 | 0.11 |
|  |  | 1980s | 0.57 | 0.09 |
|  |  | 1990s | 0.64 | 0.07 |
|  |  | 2000s | 0.49 | 0.08 |
|  |  | After 2010 | 0.07 | 0.05 |
|  | Consensual kidnap | Before 1980 | 0.25 | 0.10 |
|  |  | 1980s | 0.13 | 0.06 |
|  |  | 1990s | 0.14 | 0.05 |
|  |  | 2000s | 0.07 | 0.04 |
|  |  | After 2010 | 0.07 | 0.05 |
|  | No-kidnap | Before 1980 | 0.25 | 0.10 |
|  |  | 1980s | 0.30 | 0.08 |
|  |  | 1990s | 0.22 | 0.06 |
|  |  | 2000s | 0.44 | 0.08 |
|  |  | After 2010 | 0.86 | 0.07 |
| Husbands | Non-consensual kidnap | Before 1980 | 0.30 | 0.10 |
|  |  | 1980s | 0.33 | 0.09 |
|  |  | 1990s | 0.44 | 0.07 |
|  |  | 2000s | 0.35 | 0.07 |
|  |  | After 2010 | 0.14 | 0.07 |
|  | Consensual kidnap | Before 1980 | 0.35 | 0.11 |
|  |  | 1980s | 0.37 | 0.09 |
|  |  | 1990s | 0.32 | 0.07 |
|  |  | 2000s | 0.14 | 0.05 |
|  |  | After 2010 | 0.11 | 0.06 |
|  | No-kidnap | Before 1980 | 0.35 | 0.11 |
|  |  | 1980s | 0.30 | 0.08 |
|  |  | 1990s | 0.24 | 0.06 |
|  |  | 2000s | 0.51 | 0.08 |
|  |  | After 2010 | 0.75 | 0.08 |

**Table S2. (c)** Results of a multinomial logistic regression model examining the how report discrepancy between husbands and wives (consistent report, husband reports more consensual, wife reports more consensual) changes over marriage year cohorts (reference category: Consistent report, before 1990).

| *Report discrepancy* | *Predictors* | *Odds Ratios* | *Standard error* | *p* |
| --- | --- | --- | --- | --- |
| Husband reports more consensual | Intercept | 0.44 | 0.14 | **0.01** |
|  | 1990s | 1.03 | 0.47 | 0.94 |
|  | After 2000 | 0.27 | 0.14 | **0.01** |
| Wife reports more consensual | Intercept | 0.12 | 0.07 | **<0.001** |
|  | 1990s | 1.29 | 0.92 | 0.72 |
|  | After 2000 | 0.68 | 0.48 | 0.58 |
| Number of couples | 171 | | | |
| R^2^ / R^2^ adjusted | 0.04 / 0.03 | | | |

**Table S2. (d)** Predicted probabilities of report discrepancy between husbands and wives (consistent report, husband reports more consensual, wife reports more consensual) by marriage year cohort. Predicted probabilities were estimated based on estimated marginal means, with standard errors shown.

| *Report discrepancy* | *Marriage year cohort* | *Predicted probability* | *Standard error* |
| --- | --- | --- | --- |
| Consistent | Before 1990 | 0.64 | 0.07 |
|  | 1990s | 0.62 | 0.07 |
|  | After 2000 | 0.83 | 0.04 |
| Husband reports more consensual | Before 1990 | 0.28 | 0.06 |
|  | 1990s | 0.28 | 0.06 |
|  | After 2000 | 0.10 | 0.04 |
| Wife reports more consensual | Before 1990 | 0.08 | 0.04 |
|  | 1990s | 0.10 | 0.04 |
|  | After 2000 | 0.07 | 0.03 |

**Table S3. (a)** The contingency tables of self-reported information on marriage type in husbands and wives. The number of inconsistent reports were shown in bold.

|  | *Husband-reported* | | |
| --- | --- | --- | --- |
| *Wife-reported* | Non-consensual kidnap | Consensual kidnap | No-kidnap |
| Non-consensual kidnap | 50 | **21** | **9** |
| Consensual kidnap | **3** | 14 | **4** |
| No kidnap | **3** | **8** | 57 |

**(b)** The contingency tables of self-reported marriage type information and participant’s marriage type information reported by their mother, father, sister(s) and brother(s). The number of inconsistent reports were shown in bold; the numbers in parentheses represent the counts of females’ and males’ reports, respectively. Note that we have small sample sizes for the natal family of females because most married women were born outside the study area (do to male philopatry).

|  | *Participant’s self-report* | | |
| --- | --- | --- | --- |
|  | Non-consensual kidnap | Consensual kidnap | No-kidnap |
| *Mother’s report* |  |  |  |
| Non-consensual kidnap | 9 (2, 7) | **2 (0, 2)** | **1 (0, 1)** |
| Consensual kidnap | **3 (0, 3)** | 2 (0, 2) | **5 (0, 5)** |
| No kidnap | **4 (1, 3)** | **4 (0, 4)** | 21 (7, 14) |
| *Father’s report* |  |  |  |
| Non-consensual kidnap | 3 (1, 2) | **0** | **0** |
| Consensual kidnap | **7 (0, 7)** | 2 (0, 2) | **1 (0, 1)** |
| No kidnap | **3 (0, 3)** | **4 (0, 4)** | 18 (4, 14) |
| *Sister’s report* |  |  |  |
| Non-consensual kidnap | 13 (3, 10) | **1 (0, 1)** | **0** |
| Consensual kidnap | **5 (2, 3)** | 1 (0, 1) | **1 (0, 1)** |
| No kidnap | **3 (2, 1)** | **1 (0, 1)** | 13 (8, 5) |
| *Brother’s report* |  |  |  |
| Non-consensual kidnap | 15 (3, 12) | **6 (0, 6)** | **3 (2, 1)** |
| Consensual kidnap | **6 (2, 4)** | 4 (0, 4) | **6 (1, 5)** |
| No kidnap | **7 (1, 6)** | **10 (2, 8)** | 7 (2, 5) |
